# Time-to-response toxicity analysis as a method for drug susceptibility assessment in salmon lice

**DOI:** 10.1016/j.aquaculture.2016.08.007

**Published:** 2016-11-01

**Authors:** Greta Carmona-Antoñanzas, Joseph L. Humble, Stephen N. Carmichael, Jan Heumann, Hayden R.L. Christie, Darren M. Green, David I. Bassett, James E. Bron, Armin Sturm

**Affiliations:** a Institute of Aquaculture, School of Natural Sciences, University of Stirling, Stirling, FK9 4LA, Scotland, UK; b Marine Environmental Research Laboratory, University of Stirling, Machrihanish, Argyll, PA28 6PZ, Scotland, UK

**Keywords:** EMB, emamectin benzoate, EC_50_, median effective concentration, LC_50_, median lethal concentration, ET_50_, median effective time, LT_50_, median lethal time, PEG 300, polyethylene glycol 300, Parasite, Drug susceptibility, Emamectin benzoate, Sea lice, Salmon delousing agent

## Abstract

The salmon louse *Lepeophtheirus salmonis* (Krøyer, 1837) is an ectoparasite causing infections of wild and farmed Atlantic salmon (*Salmo salar* L.) in the Northern hemisphere. While *L. salmonis* control at commercial mariculture sites increasingly employs non-medicinal approaches, such as cage designs reducing infection rates and biological control through cleaner fish, anti-parasitic drugs are still a requirement for effective fish health care. With only a limited range of salmon delousing agents available, all of which have been in use for more than a decade, drug resistance formation has been reported for different products. Successful resistance management requires reliable susceptibility assessment, which is usually achieved through *L. salmonis* bioassays. These tests involve the exposure of parasites to different drug concentrations and require significant numbers of suitable *L. salmonis* stages. The present study reports an alternative bioassay that is based on time-to-response toxicity analyses and can be carried out with limited parasite numbers. The assay determines the median effective time (ET_50_), i.e., the time required until impaired swimming and/or attachment behaviour becomes apparent in 50% of parasites, by conducting repeated examinations of test animals starting at the time point where exposure to a set drug concentration commences. This experimental approach further allows the estimation of the apparent drug susceptibility of individual *L. salmonis* by determining their time to response, which may prove useful in experiments designed to elucidate associations between genetic factors and the drug susceptibility phenotype of parasites. Three laboratory strains of *L. salmonis* differing in susceptibility to emamectin benzoate were characterised using standard 24 h bioassays and time-to-response toxicity assays. While both the median effective concentration (EC_50_) and the ET_50_ showed variability between experimental repeats, both types of bioassay consistently discriminated susceptible and drug-resistant *L. salmonis* laboratory strains.

**Statement of relevance:**

Infections by sea lice cause significant costs to the global salmon farming industry, which have been estimated to exceed €300 million per year worldwide. Control of sea lice still relies to a significant extent on chemical delousing; however, chemical control is threatened by resistance formation. Resistance can be combated by rotation between different drugs and strategic implementation of non-medicinal strategies. However, resistance management requires reliable and feasible methods of susceptibility assessment.

The present study is a technical note introducing a novel approach to susceptibility assessments in sea lice. The method can be applied in susceptibility assessments on farms, where it offers the advantage of a reduced requirement of parasites for testing. In addition, the novel method allows deriving the times of parasite require to show a response after drug treatment has started, thus providing a variable characterizing the drug susceptibility phenotype of individual parasites. Accordingly, the bioassay approach presented here will be useful for studies aiming at unravelling the genetic determinants of drug resistance.

## Highlights

• Bioassays are used to assess drug susceptibility in the salmon louse, a commercially relevant fish parasite
• A novel bioassay based on time-to-response toxicity analyses requires fewer test animals than standard bioassays.
• The novel bioassay further allows characterisation of the drug susceptibility phenotype in individual parasites.

## 1 Introduction

Sea lice (Caligidae: Copepoda) are marine fish ectoparasites feeding on the host's mucus and skin tissues (Boxaspen, 2006). Sea louse infections of farmed Atlantic salmon (*Salmo salar*) mostly involve *Lepeophtheirus salmonis* (salmon louse) and *Caligus elongatus* in the Northern hemisphere, and *Caligus rogercresseyi* in Chile (Costello, 2006). Sea louse control on salmon farms relies mainly on the use of veterinary drugs, supplemented by farm management measures (Costello, 2009) and non-medicinal approaches such as the deployment of cleaner fish (Sayer et al., 1996) and modified cage designs reducing the likelihood of infection (Stien et al., 2016).

Only a restricted range of licensed anti-sea louse drugs are currently available (Burridge et al., 2010). The continued use of the same or a few types of control agents, however, can favour the development of drug resistance in parasites (Denholm et al., 2002). Drug resistance is well documented in *L. salmonis*, and compounds for which loss of efficacy has been reported include the organophosphates dichlorvos and azamethiphos (Jones et al., 1992, Kaur et al., 2015, Roth et al., 1996), the pyrethroid deltamethrin (Sevatdal and Horsberg, 2003), the non-specific oxidising agent hydrogen peroxide (Treasurer et al., 2000) and the macrocyclic lactone emamectin benzoate (EMB) (Lees et al., 2008). Resistance to different drugs has further been found in *C. rogercresseyi* (Agusti et al., 2016, Bravo et al., 2010). Accordingly, there is an urgent need for efficient resistance prevention and management strategies in sea lice (Aaen et al., 2015).

A key requirement for such effective sea louse control strategies is an accurate assessment of the drug susceptibility status of sea louse populations. Such assessments are usually achieved by conducting bioassays, which typically involve subjecting batches of preadult or adult parasites to a dilution series of the drug (Sevatdal and Horsberg, 2003, Westcott et al., 2008). The internal exposure of an aquatic organism taking up a toxicant from water will increase both with increasing toxicant concentration and increasing length of exposure. Traditional aquatic bioassays typically employ different toxicant concentrations to achieve gradually varied exposures, while keeping the length of the exposure period constant. Results are expressed as the median lethal or median effective concentration (LC_50_, EC_50_), i.e., the concentration theoretically causing a toxic effect in 50% of the tested population. In an alternative approach called time-to-response toxicity analysis, gradual exposure levels are achieved by combining a fixed toxicant concentration with variable exposure periods. In this approach, the susceptibility of the population to the toxicant is expressed as the median lethal or effective time (LT_50_, ET_50_) (Robertson and Preisler, 1991). Time-to-response bioassays have been used in susceptibility assessments of terrestrial arthropod pests (Robertson and Preisler, 1991) and ecotoxicity studies in aquatic invertebrates and fish (Pascoe and Edwards, 1989, Rand, 2008).

The availability of sea louse stages suitable for bioassays can be restricted at production sites. Alternative bioassays involving only one drug concentration and a fixed exposure period have been proposed to allow for drug susceptibility assessment under such circumstances (Helgesen and Horsberg, 2013a, Whyte et al., 2013). While single-dose bioassays can be highly useful as a tool in fish health management, their ability to resolve susceptibility differences between parasite populations is by design limited. Time-to-response toxicity analyses could provide a complementary approach allowing characterisation of the susceptibility status at greater depth when sea louse availability is restricted. In addition to supporting veterinary decisions on fish farms, drug susceptibility assessments in sea lice are central to experimental plans aiming at identifying genetic determinants of drug resistance, which often require the determination of the susceptibility phenotypes of individuals (Besnier et al., 2014). Differentiation between susceptible and resistant parasites has been previously achieved by rating toxic responses following exposure to a diagnostic drug concentration for a set time period (Ljungfeldt et al., 2014). Using a similar approach, but additionally implementing repeated observations to determine the time to response for individual parasites, would permit a more graduated characterisation of the drug susceptibility phenotype than achievable with a test design employing a one concentration/one exposure time cut-off criterion to define resistance/susceptibility.

The aim of the present study was to investigate the potential of time-to-response toxicity analyses as an alternative approach to conducting sea louse drug sensitivity assessments. Time-to-response toxicity analyses were compared to standard bioassays with respect to their ability to differentiate between well-characterised laboratory strains of *L. salmonis* showing different degrees of resistance to the salmon delousing agent EMB.

## 2 Materials and methods

### 2.1 Salmon louse (*L. salmonis*) strains and husbandry

Three *L. salmonis* laboratory-maintained strains established from field isolates of egg strings without further selection in the laboratory were used in this study. The drug-susceptible strain IoA-00 (previously called “S″) (Heumann et al., 2012) was established in 2003 from a Scottish farm site where no chemical control agents other than hydrogen peroxide had been used. The EMB-resistant strain IoA-01 (previously called “PT” or “R”) (Heumann et al., 2012, Heumann et al., 2014) and the multi-resistant strain IoA-02, which is hyposensitive to both EMB and deltamethrin, were created in December 2008 and September 2011, respectively, from other Scottish sites where there had been reports of variable treatment efficacies. These strains have since been cultured under identical laboratory conditions using Atlantic salmon as host fish, as described in detail elsewhere (Heumann et al., 2012). To propagate cultures, *L. salmonis* egg strings were collected from gravid females and allowed to hatch and develop to infective copepodids, which were used to inoculate tanks containing fresh host fish. To collect *L. salmonis*, host fish were either euthanised under a UK Home Office approved Schedule 1 method, or anaesthetised with 100 mg L^− 1^ 2-phenoxyethanol (99%; Sigma-Aldrich, Dorset, UK) in seawater for 3 min. Previous experiments assessing the effect of anaesthesia on bioassay results did not find significant differences between the two sea lice collection methods (data not shown). Parasites were removed from fish into clean aerated seawater using fine forceps, and fish were transferred into clean seawater with aeration for recovery. Infection rates were maintained at levels compatible with good fish welfare according to MERL Good Laboratory Practise (GLP) Standard Operating Procedure (SOP) protocols. All laboratory infections were carried out under UK Home Office licence and appropriate veterinary supervision.

### 2.2 Standard bioassays

Experiments used adult male or preadult II female *L. salmonis*. After collection from host fish, parasites were allowed to recover for 2–4 h in filtered aerated seawater at 12 °C before use in bioassays. Exposures were performed in a temperature-controlled incubator set to 12 °C, using 150 mL plastic Petri dishes holding 70 mL of exposure solutions and containing ten sea lice. EMB (technical grade, a gift from MSD Animal Health) was solubilised using polyethylene glycol of a number average molecular weight (M_n_) of 300 (PEG 300, pH. Eur., Merck Chemicals, UK) before being diluted in seawater. Exposure solutions contained a final concentration of 0.05% (*v*/v) PEG 300. Each test comprised a geometrical dilution series of EMB of at least five concentrations in addition to seawater and solvent controls, the latter containing 0.05% (v/v) PEG 300. Sea lice were assigned to treatments randomly. At least two replicate Petri dishes were used per combination of strain and drug or control treatment. After 24 h of exposure, sea lice were visually examined and rated according to their attachment and mobility behaviour. Prior to rating, beakers were re-labelled with codes by laboratory staff not involved in the recording of experimental outcomes to allow for observer-blinded rating. In experiments conducted in 2011 and before, salmon lice were rated as normally motile (unaffected) or immotile (affected) upon visual examination and stimulation with a fine brush (Heumann et al., 2012). Later experiments used rating criteria initially proposed by Sevatdal and Horsberg (2003) and Westcott et al. (2008) and modified by Igboeli et al. (2012), where parasites are rated “live” when firmly attached to the surface of the Petri dish or swimming normally, “weak” when swimming irregularly and failing to attach to surfaces firmly (animals may attach briefly, but dislodge again instantly), “moribund” when incapable of swimming away or attaching (animals may twitch appendages or move uncoordinatedly in a close circle) and “dead” when showing no movements in extremities, gut or other organs as apparent from examination under a microscope. Using this set of criteria, parasites were considered unaffected when rated “live” or “weak” and affected when rated “moribund” or “dead”. Bioassays were considered invalid if > 10% of parasites in control treatments were deemed affected.

### 2.3 Time-to-response bioassays

Sea lice were exposed for 24 h to 400 μg L^− 1^, 800 μg L^− 1^ or 1200 μg L^− 1^ of EMB, using similar general procedures and involving the same types of controls and levels of replication as described in Section 2.1.2. At set time intervals throughout the exposure period, parasites were visually examined and rated as described in Section 2.1.2. Typically, this involved hourly examinations for the beginning of the exposure period (1–15 h after addition of toxicants) followed by further examinations at 18, 21 and 24 h. Experimentation thus involved repeated examination of the same sea lice. In time-to-response bioassays, the main factor controlling toxic response is exposure time, which cannot be concealed to the experimenter by coded labelling. To ensure impartial rating in these assays, animals were rated by a second observer unaware of the nature of the experiment at selected time points. Ratings of immotile parasites per Petri dish never differed by more than one between observers. All other experimental conditions were identical to those described in Section 1.1.2 for the standard bioassay.

### 2.4 Statistics

In standard bioassays, median effective concentrations (EC_50_) of EMB and 95% confidence intervals were generally calculated by probit analysis using the statistical programme Minitab 16.1.0 (Minitab Inc.). In cases where data were not suitable for probit analysis, the trimmed Spearman–Karber method was used to derive the EC_50_ and 95% confidence limits (Hamilton et al., 1977). In time-to-response bioassays, median effective times (ET_50_) and 95% confidence intervals were estimated using the survival time module of Minitab, assuming a log-normal distribution. In all tests associated to the above estimations, results were considered significant if the probability value (*P*) was < 0.05.

## 3 Results

### 3.1 Standard bioassay

**Fig. 1:**
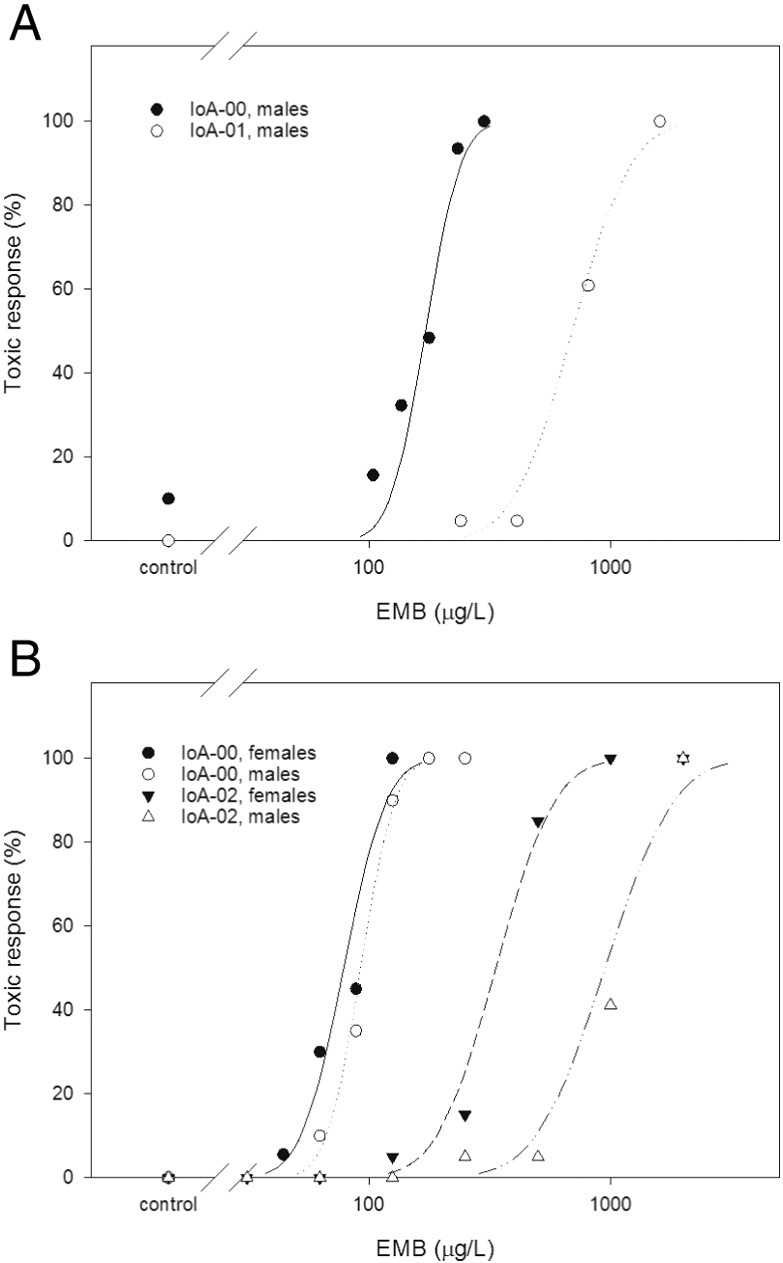
Standard emamectin benzoate (EMB) bioassay with *Lepeophtheirus salmonis*. Symbols represent the average behavioural toxic response of at least two batches of ten animals, recorded after 24 h of exposure to EMB in plastic Petri dishes. A, EMB toxicity in salmon louse strains IoA-00 and IoA-01. B, EMB toxicity in strains IoA-00 and IoA-02.

Three laboratory-maintained salmon louse strains differing in susceptibility to EMB were used in this study. When applied at concentrations > 100 μg L^− 1^, EMB induced behavioural toxic responses in strain IoA-00 in a concentration-dependent manner (Fig. 1). Higher EMB concentrations were required to provoke similar toxic responses in strains IoA-01 and IoA-02 (Fig. 1). Strain differences in EMB susceptibility are illustrated by median effective concentrations (EC_50_) obtained in different experiments, with estimates for the EC_50_ in adult males ranging from 74.3 to 159.3 μg L^− 1^ for IoA-00, from 553 to 780 μg L^− 1^ in IoA-01 and 445 to 675 μg L^− 1^ in IoA-02 (Table 1). With strain IoA02, preadult II females were significantly more susceptible to EMB than adult males. By contrast, gender differences in EMB susceptibility were not significant in strain IoA-00 (Table 1).

### 3.2 Time-to-response bioassay

**Fig. 2:**
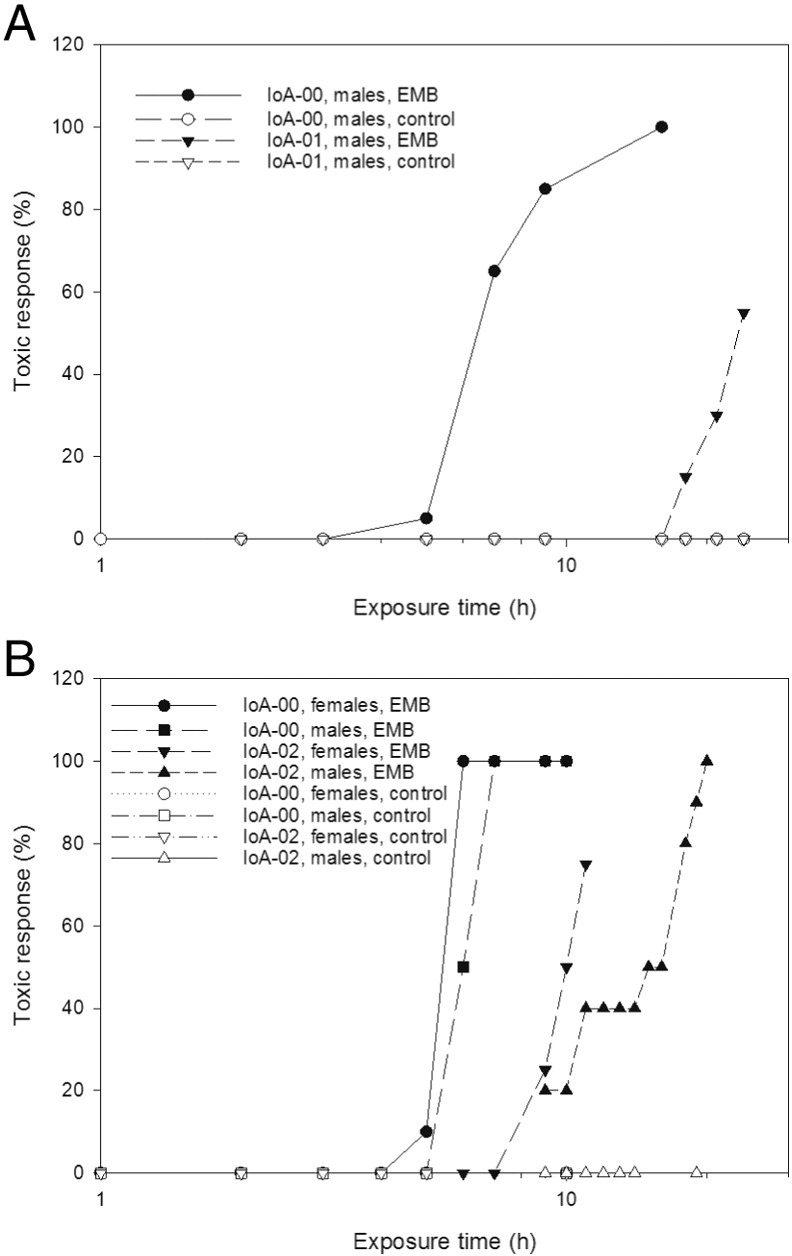
Time-to-response bioassay with *Lepeophtheirus salmonis.* Symbols represent the average behavioural toxic response of at least ten animals. Salmon lice were exposed to 800 μg L^− 1^ of emamectin benzoate (EMB) for a total period of 24 h, during for which repeated observations of parasite motility and attachment behaviour were made. A, EMB toxicity in salmon louse strains IoA-00 and IoA-01; B, EMB toxicity in strains IoA-00 and IoA-02.

To investigate the potential of time-to-response salmon louse bioassays to assess strain and gender differences in EMB susceptibility, experiments were first conducted using an exposure concentration of 800 μg L^− 1^ (Fig. 2). Apparent response levels in exposed parasites increased in a time-dependent fashion, allowing the median effective time (ET_50_) to be derived for the populations tested (Table 2). While ET_50_ values determined at an exposure level of 800 μg L^− 1^ EMB showed some variation between experiments, bioassays with male salmon lice consistently found greater ET_50_ values in strains IoA-01 and IoA-02 than strain IoA-00 (Table 2). During exposure to 800 μg L^− 1^ EMB, response levels in the IoA-00 strain increased very rapidly after first signs of toxicity became apparent, with few or no partial responses being observed at set examination times (Fig. 2). This precluded the estimation of 95% confidence limits in some experiments (Table 2). Subsequent time-to-response experiments employed EMB levels of 400 or 1200 μg L^− 1^. Changing the EMB exposure concentration within the above limits had little effect on the time course of toxicity in the IoA-00 strain, as apparent from ET_50_ values showing overlapping 95% confidence intervals (Table 2). Similarly, ET_50_ values for IoA-01 males were similar between trials using 800 and 1200 μg L^− 1^ of EMB (Table 2). By contrast, ET_50_ values for IoA-02 females at 400 μg L^− 1^ were significantly greater than at 800 μg L^− 1^ EMB. In exposures with 400 μg L^− 1^ EMB, no ET_50_ could be determined for IoA-02 males, as these salmon lice failed to show apparent signs of toxicity within the 24 h experiment (Table 2).

## 4 Discussion

The assessment of drug susceptibility of salmon louse populations causing farm infections is essential to provide information to fish health professionals and aid the identification of effective therapeutic options. Susceptibility assessments are commonly based on the performance of salmon louse bioassays, which involve the exposure of batches of parasites to drug concentrations forming a geometrical series. While salmon louse bioassays are well standardised and field-tested (Sevatdal and Horsberg, 2003, Westcott et al., 2008), their requirement for significant numbers of test animals can be problematic in situations where the availability of suitable parasite stages is restricted. A typical bioassay with five test concentrations and one control and involving duplicate experimental and control treatments with ten animals each will require 120 parasites. By comparison, only 40 parasites are required in time-to-response bioassay at the same level of replication. While the time-to-response bioassay described here is more labour intensive than single-dose bioassays involving a single exposure time (Helgesen and Horsberg, 2013a, Whyte et al., 2013), the time-to-response bioassay characterises the drug susceptibility phenotype of the given parasite population at greater resolution. Thus, the time-to-response bioassay can be expected to complement existing methods of drug susceptibility assessment in salmon lice with deployment of the new bioassay depending on context of the study. In addition to the potential for implementation of this novel bioassay in the assessment of farm site infections, the time-to-response bioassay is capable of providing estimates of the drug susceptibility of individual parasites, which suggests its usefulness in the context of analysing molecular responses of sea lice to anti-parasitic drugs.

While salmon lice parasitising EMB-treated fish experience drug exposure both through contact with and ingestion of mucous and skin tissues, parasites are exposed solely through the aqueous route in 24 h bioassays, complicating the prediction of treatment efficiencies from bioassay findings. In an earlier study, EMB medication (50 μg kg^− 1^, seven days) of experimentally infected salmon resulted in 98% salmon lice clearance for strain IoA-00 parasites by day 21 after the start of treatment cycle but had no effect on IoA-01 salmon lice (SARF, 2012). Similarly, no clearance of strain IoA-02 was observed from a standard EMB treatment cycle (data not shown). Accordingly, the levels of resistance of parasite strains used in this study are clinically relevant. The fact that the time-to-response bioassay described in this work is able to differentiate between the above EMB-resistant strains and a susceptible reference strain suggests the usefulness of the time-to-response bioassay for susceptibility assessments.

Salmon louse bioassays have been initially defined using a parasite behaviour rating scale of three categories (Sevatdal and Horsberg, 2003, Westcott et al., 2008). According to this scale, animals are rated “live” at the end of the test when they attach firmly to the Petri dish or show normal locomotive behaviour, such as swimming in a straight line. By contrast, “moribund” salmon lice show impaired motility behaviours, ranging from irregular swimming to inability to move, as well as weak adhesion. Parasites are rated “dead” if they show no movement in the extremities or the gut (Sevatdal and Horsberg, 2003, Westcott et al., 2008). More recently, different groups have adopted a four category rating scheme, involving a more narrow definition of “moribund”, which now includes only immotile parasites still showing twitching of appendages but not irregular swimmers, and the new category “weak”, which comprises parasites displaying irregular swimming or weak attachment to the Petri dish wall (Igboeli et al., 2012, Saksida et al., 2013). In the present study, this four criteria scale was adapted in experiments carried out in 2012 and thereafter, while earlier experiments used the three-category scale (Sevatdal and Horsberg, 2003, Westcott et al., 2008). The change in rating criteria had little effect on the outcome of most bioassays but markedly reduced the number tests that were invalid due to excessive response levels in controls, which were mostly due to parasites showing weak attachment behaviour but only slightly impaired swimming. The present investigation included standard and time-to-response bioassays conducted with both methodologies, so that changes in rating scale do not affect the study's findings.

EMB EC_50_ values obtained in different experiments with male parasites ranged from 74.3 to 159.3 μg L^− 1^ for IoA-00 and 553.0 to 780.3 μg L^− 1^ for IoA-01. The lower EMB EC_50_ of 289.0 μg L^− 1^ obtained for IoA-01 males in a previous study has been excluded from data considered in this study, as DMSO was used as solvent carrier in that study (Heumann et al., 2014), which may have influenced toxicity (Tanneberger et al., 2010). While results obtained in the current study illustrate variability among EC_50_ estimates, standard bioassays consistently differentiated the EMB-resistant strains IoA-01 and IoA-02 from the susceptible strain IoA-00. Paralleling the results obtained in the present study, Espedal et al. (2013) observed consistent strain differences in EMB susceptibility despite between-test variability in absolute EMB bioassay estimates. Similar to standard bioassays, the time-to-response bioassay introduced in the present report displays a certain degree of variation in ET_50_ values between repeated experiments. Factors that might be responsible for the variability observed in this study include seasonal effects, as well as variability in the actual test concentrations of EMB, known for its hydrophobicity. An earlier study from this laboratory detected 49.7% of a nominal EMB concentration of 200 μg L^− 1^ 3 h after the start of exposures (Carmichael et al., 2013). Similarly, in another study (Helgesen and Horsberg, 2013b), 48.9% of an nominal concentration of 150 μg L^− 1^ EMB was detected in the seawater phase at the end of 24 h salmon louse bioassays performed in high-density polyethylene dishes. In the same study, recoveries were slightly less in tests using glass dishes (41.9%) and substantially inferior when using Teflon™ or silanised glass containers (27.4% and 24.0%, respectively) (Helgesen and Horsberg, 2013b).

### 4.1 Conclusion

In conclusion, the present study describes a novel salmon louse bioassay based on time-to-response analyses, which requires less test animals than current standard salmon louse bioassays. Our results indicate that time-to-response bioassays are powerful tools to assess the susceptibility of salmon louse populations to control agents such as EMB, particularly in situations where the availability of test animals is restricted. A further advantage of this novel bioassay is its ability to provide estimates of the drug susceptibility of individual parasites, which is expected to be useful in the context of analysing molecular responses of salmon lice to anti-parasitic drugs.

## Figures and Tables

**Table 1:** Susceptibility of *Lepeophtheirus salmonis* strains to emamectin benzoate. The toxicity of EMB to defined parasite stages of different laboratory-maintained strains was determined in 24 h bioassays. Results are expressed as EMB median effective concentrations (EC_50_), determined by probit analysis except where noted otherwise.

Strain	Sex/stage	Response rate in controls	EC_50_ (μg L^− 1^) (95% confidence limits)	Date of experiment
IoA-00	Male, adult	10%	159.3 (145.4–175.6)^1^	01/2011
	Male, adult	0%	74.3 (59.0–94.1)^2^	05/2011
	Male, adult	0%	92.4 (83.2–106.7)	03/2015
	Female, preadult II	0%	78.6 (70.0–88.3)	03/2015
IoA-01	Male, adult	0%	695.5 (583.5–841.1)^1^	11/2010
	Male, adult	0%	780.3 (642.9–967.4)^2^	05/2011
	Male, adult	2.5%	553.0 (486.1–624.7)	07/2011
IoA-02	Male, adult	0%	949.8 (767.8–1204.8)^3^	11/2011
	Male, adult	0%	675.0 (618.0–738.0)^4^	10/2014
	Female, preadult II	0%	335.3 (277.0–409.4)^3^	11/2011
	Female, preadult II	0%	355.1 (289.9–521.1)	03/2015

1 Data shown in Fig. 1A.

2 Data from Carmichael et al. (2013).

3 Data shown in Fig. 1B.

4 Trimmed Spearman–Karber statistics.

**Table 2:** Toxic response of *Lepeophtheirus salmonis* strains to emamectin benzoate in time-to-response bioassays. Toxicity is expressed as the median effective time (ET_50_).

Date	EMB (μg L^-1^)	ET_50_ (h) (95% confidence limits)				
		IoA-00		IoA-01	IoA-02	
		Males	Females	Males	Males	Females
12/2011	800	5.86^1^	4.03 (3.67–4.43)	21.36 (16.82– 27.13)		
07/2012^2^	800	6.77 (6.08–7.54)		28.46 (15.94–50.84)		
07/2012	1200	5.72 (5.07–6.44)		25.66 (17.49–37.65)		
03/2015^3^	800	6.00^1^	5.08^1^		13.18 (10.51–16.51)	9.98 (8.99–11.08)
07/2015	400	6.82 (6.33–7.35)	5.21 (4.68–5.81)		> 24^4^	16.50 (13.91–19.56)

1 Data do not allow calculation of 95% confidence limits.

2 Data set shown in Fig. 2A.

3 Data set shown in Fig. 2B.

4 Data do not allow calculation of ET_50_.
